# Predicting the Mutating Distribution at Antigenic Sites of the Influenza Virus

**DOI:** 10.1038/srep20239

**Published:** 2016-02-03

**Authors:** Hongyang Xu, Yiyan Yang, Shuning Wang, Ruixin Zhu, Tianyi Qiu, Jingxuan Qiu, Qingchen Zhang, Li Jin, Yungang He, Kailin Tang, Zhiwei Cao

**Affiliations:** 1 School of Life Science and Technology, Tongji University, Shanghai, China; 2 Department of Computational Regulatory Genomics, CAS-MPG Partner Institute for Computational Biology, Shanghai Institutes for Biological Sciences, Chinese Academy of Sciences, Shanghai, China; 3 Shanghai Center for Bioinformation technology, Shanghai, China; 4 The State Key Laboratory of Cancer Biology, School of Pharmacy, Department of Biopharmaceutics, The Fourth Military Medical University, Xi’an, China; 5 Ministry of Education Key Laboratory of Contemporary Anthropology, School of Life Sciences and Institute of Biomedical Sciences, Fudan University, Shanghai, China

## Abstract

Mutations of the influenza virus lead to antigenic changes that cause recurrent epidemics and vaccine resistance. Preventive measures would benefit greatly from the ability to predict the potential distribution of new antigenic sites in future strains. By leveraging the extensive historical records of HA sequences for 90 years, we designed a computational model to simulate the dynamic evolution of antigenic sites in A/H1N1. With templates of antigenic sequences, the model can effectively predict the potential distribution of future antigenic mutants. Validation on 10932 HA sequences from the last 16 years showing that the mutated antigenic sites of over 94% of reported strains fell in our predicted profile. Meanwhile, our model can successfully capture 96% of antigenic sites in those dominant epitopes. Similar results are observed on the complete set of H3N2 historical data, supporting the general applicability of our model to multiple sub-types of influenza. Our results suggest that the mutational profile of future antigenic sites can be predicted based on historical evolutionary traces despite the widespread, random mutations in influenza. Coupled with closely monitored sequence data from influenza surveillance networks, our method can help to forecast changes in viral antigenicity for seasonal flu and inform public health interventions.

The seasonal influenza virus is well known for its rapid mutation rate and constant antigenic changes, which causes a major and persistent challenge to public health. To better understand these changes, previous studies have established evolutionary models to trace back the genomic variations and epidemiological dynamics at genome level^1^,^2^. Meanwhile, the rapid accumulation of high-quality genome sequences has provided new opportunities to analyse virus spread and phylodynamics based on current and past influenza genomes aiming for better preventive measures^3^,^4^. Recently, several computational studies proposed effective methods to help in recommending vaccine strains as early as possible from known genomic sequences^5^,^6^,^7^.

The above efforts were informative, but the spread of seasonal influenza remains a challenge despite various efforts of vaccination strategies. Several factors may hinder the efficacy of the current vaccination strategies. At present WHO usually recommends strains which have arisen in the past as vaccine strains. Despite of the rapid response from WHO in making recommendations, at least six to eight months are required for industrial production and market distribution of the vaccine. When the vaccines are applied seasons later, the proposed strains may no longer be prevalent in the community, reducing the efficacy of the vaccine. Most importantly, a large amount of new mutants, especially within the epitope regions of major HA antigens, will likely have emerged across the seasons and may evade the protection provided by the WHO-recommended vaccines. Only when the future distribution of new epitope mutants is obtained for major antigens, can better preventive measures be achieved. Although a wealth of genomic data has been accumulated, extensive data of antigenic sequences has not yet been used to predict future mutant profiles. The question we sought to address here is whether the distribution profiles of the new antigenic variants are predictable in upcoming seasons.

In our study, the HA protein sequences of the A/H1N1 virus were used as an example to demonstrate how to achieve the above goals. We demonstrated that our model also perform well with A/H3N2 data. Our results revealed that the future mutant profiles of the HA antigen sites are predictable, including those dominant antigenic sequences.

## Results

### Model construction to compute mutating distribution for HA antigenic sites

Seasonal influenza is hypothesized to escape host immune responses by gradual genetic evolution or a neutral network until a dominant strain with higher fitness emerges^8^. Then, the offspring of this beneficial mutant will spread throughout the population and cause a sudden shift in phenotype^9^. Above hypothesis motivated us to compute the potential distribution of future mutants from seeding or template sequences. The assumptions of our model include the following: 1) the major antigen of HA experiences greater evolutional pressure compared with other proteins in the virus, and thus deserve an antigen-specific evolutional model instead of a model at genome level; 2) residual diversities at different positions of antigenic sites often imply different adaptive abilities, such as contact transmissibility^10^ and immune-escaping ability, while the adaptive ability might be partially inferred from the historical trace of antigenic positions; and 3) the final dominance of an antigenic mutant may be related not only to its inherent adaptive ability, but also its population. The bigger population of an antigenic sequence accumulated, the more likely it may survive the natural selection.

**Figure 1:**
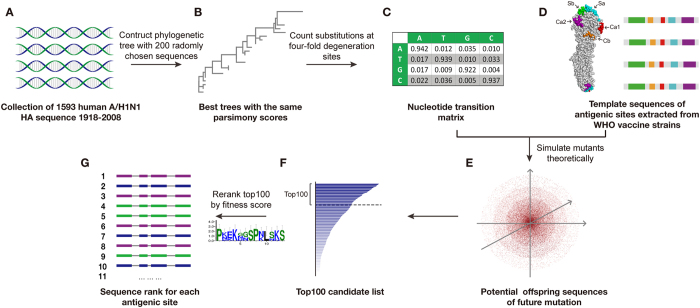
Overview of our model to calculate the mutating distribution for the HA antigenic sites. Steps (**A–C**) illustrate the construction of the nucleotide transition matrix for HA antigens. Steps (**D**–**F**) present the mutant simulation and selection for the template sequences. In step (**G**), the top 100 mutants are re-ranked according to potential dominance likeliness score.

Figure 1 presents the workflow of the model. In steps A to C, a nucleotide transition matrix was generated for HA antigen according to 90 years of historical training data. To construct the phylogenetic trees, representative HA nucleotide sequences were randomly chosen from training data based on geographic and temporal ranges. Steps D to G represent the mutation-selection ranking model for the template sequences. The epitope regions of HA protein have been extensively studied for H1N1 and well characterized as five antigenic sites. In this study, we chose a large epitope area with 11 residues for Ca1, 8 for Ca2, 6 for Cb, 13 for Sa and 12 for Sb^11^,^12^,^13^,^14^. After simulation and redundancy removal, the top abundant sequences with big population were selected for further ranking according to dominance likeliness denoted by both theoretical population and adaptive ability. Different cutoffs of relative abundance were tested for the model performance, as illustrated in Supplementary Figure S1. The top 100 were tentatively chosen given that most of the dominant sequences will not be missed under this cutoff. Normally, the top 100 cutoff can cover 50 to 80% of simulated sequences before redundancy removal and 0.05 to 0.1% of non-redundant sequences simulated for each site.

Sampling was done for the representative sequences to construct phylogenetic tree and results suggest no statistical differences between simulated trees. A background model was also constructed based on the simulation of random mutations as a control. The mutation rate from a specific DNA base to any other was equally set to 0.25 to eliminate evolutionary pressure differences. The results of this random model were all zero, supporting the validity of our mutation model.

### Model validation using posterior observed data

The model was evaluated via data from 1999 to 2014 using WHO-recommended vaccine strains as templates. Over the 16 years, a total of four vaccine strains were primarily recommended by WHO: 1) the A/Beijing/262/1995 (H1N1)-like strain was proposed in February 1999 for the Northern hemisphere; 2) the A/New Caledonia/20/1999 (H1N1)-like strain was recommended in October 1999 for the Southern hemisphere; 3) the A/Solomon Island/3/2006 strain was given in September 2006; and 4) the A/California/07/2009 strain was suggested in April 2009.

Two measurements, type coverage and strain coverage, were defined for the model evaluation. The difference between them is whether the sequence redundancy is removed among the same group of antigenic sites of collected strains (see MATERIALS AND METHODS). Thus the strain coverage is expected to be more indicative than type coverage in evaluating the infection spread within a community. Prediction results with low type coverage but high strain coverage often include dominant and widely distributed antigenic mutants.

**Figure 2:**
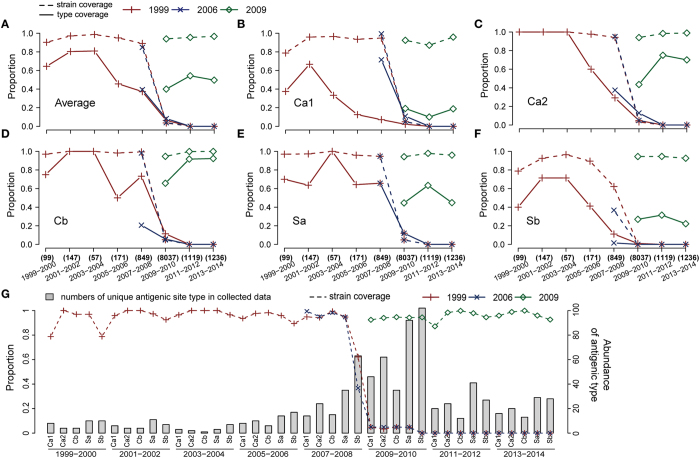
The type coverage and strain coverage during the entire validation period from 1999 to 2014. The results were grouped every two years using three different template strains announced in 1999, 2006 and 2009. (**A**) The average results of the five antigenic sites representing the overall epitope areas on the HA protein. The Y-axis on the left indicates the type coverage (solid line) and strain coverage (dashed line) in proportion. The X-axis indicates the evaluation period. (**B**–**F**) Prediction results for each individual site. (**G**) The strain coverage of the prediction profile with three template strains and five sites indicated in one graph. The Y-axis on the right indicates the number of antigenic types in the bar plot.

Figure 2 displays the type coverage and strain coverage across 16 years for each individual antigenic site. For clearer illustration, all antigenic sequences during the 16 years are grouped by two-year intervals and the detailed results from the 1995 template are presented separately in Supplementary Table S1. As observed in Fig. 2, the type coverage tends to drop with time for both the combined and individual sites. This could be explained by the increasingly accumulated sequence data and mutant types reported over time. We also observed a diversity peak of each antigenic site during 2009–2010 (Fig. 2G), supporting the critical adaptation phase to new host^4^. From 2009 when a new type of “swine flu” emerged, all predictions based on previous templates become ineffective. However, this failure was soon rescued by template updating. Overall, the strain coverage are maintained at above 94% on average of the five sites with proper template replacement for the past 16 years despite of the erratic curve of type coverage (Fig. 2G). This finding suggests that although a vast number of mutants continually emerge, over 90% of new antigenic sequences in reported strains fall within the predicted profiles.

Different mutants often co-exist at a given time, but their fitness abilities to host pressure vary significantly. It is desirable to investigate how well those dominant antigenic mutants observed in the community can be predicted by our model. Table 1 presents the predicted ranking of five antigenic sites in those globally dominant strains. In general, 96% (24/25) of antigenic sites in the dominant strains are located within the top 100 profiles, and 92% (23/25) are located within the top 50 lists for each site. Interesting finding is that some antigen sites of dominant strains seldom change within an epidemic cycle divided by vaccine strain replacement, such as Ca2 and Cb, whereas others change more frequently. The ability of our method to successfully predict the top few dominant mutants of antigenic sites suggests an advance beyond existing methods that could be important for informing preventive strategies.

### Model performance of different template sequences

As being indicated, WHO recommended vaccine strains are good templates to predict future mutant profiles at antigenic sties. However, it is highly desirable to assess the model application during emergency outbreak when WHO vaccine strain has not become available yet. In recent history of A/H1N1, April of 2009 represents a critical time when a new type of “swine flu” virus with the host transferring ability became pandemic in human community. Compared with the other seasonal H1N1 influenza, the diversification and spread of “swine flu” were well characterized during the early outbreak^15^ and thus presented an opportunity to investigate the template influence in emergent outbreak.

We tested two earliest reported sequences and another two abundant representatives, each from the top two abundant epitope clade covering five sites in the month of April 2009. The clades are defined as clusters of sequences sharing the same epitopes (combined by five antigenic sites). Then these clades are ranked by their sequence abundancy (see Supplementary Table S2). Coincidently, the earliest reported strain A/California/04/2009 shares the exactly same epitope sequences with the WHO recommended strain A/California/07/2009, which also lies in the largest epitope clade in the prediction results. Compared with A/California/04/2009 (both the earliest and in the most abundant clade), the predicted type coverage and strain coverage for A/Mexico/3955/2009 (early but not abundant) were considerably reduced (see Supplementary Table S3). The likely reason could be that the early strains may still be in the process of host adaptation without enough fitness ability. However, the predictions from A/Ohio/07/2009 (the second most abundant) performed similarly well as the A/California/07/2009, suggesting the multiple possibilities of template choosing (see Supplementary Table S3). Finally, the mutant profiles at antigenic sites of A/H1N1 influenza virus are predicted based on 2014 template in Supplementary Table S4 for future validation after 2015.

### Model application to A/H3N2

We assessed the broader applicability of our method by applying it to the human influenza A/H3N2. When the model was tested by data from 2002 to 2014, an average strain coverage achieved 92.97% as being presented in Supplementary Figure S2. Moreover, 74.1% of the antigenic sites in dominant epitopes are located within the top 100 profiles, and 70.4% fall in the top 50 profile for each site (see Supplementary Table S5). Corresponding type coverages and strain coverages are presented in detail in Supplementary Figure S3 and Table S6. It is worth noting that more frequent vaccine strains are recommended by WHO for A/H3N2 compared with A/H1N1. So does to the template replacement. This difference may result from the higher rate of adaptive evolution for H3N2^16^. Our predictions for H3N2 are thus comparable with H1N1, although slightly less effective in the B site, which is likely due to the different preferences for genetic changes observed in this region^17^. Therefore, our model is applicable to different influenza virus subtypes.

## Discussion

In spite of the latest computational technology to facilitate vaccine design, current preventive strategies can only evaluate and recommend those influenza variants that have arisen in the past. Predicting future profiles of new antigenic mutants would significantly improve the efficacy of current measures through expecting the upcoming antigenicity. In this study, a computational model was designed to simulate the future distribution of new antigenic mutants based on the evolutionary footprints at antigenic sites. Our results provide strong evidence that the profile of future antigenic variants of the HA antigen can be predicted along their own evolutionary trajectories despite wide, fast and constant changes.

Template selection plays an important role in predicting future dominant mutants. The most effective and desirable templates would be those epitopes being able to produce more offspring to survive the natural selection in community. The vaccine strains proposed by the WHO are based on information regularly provided by the WHO Global Influenza Surveillance Network (GISN), combining retrospective antigenic, epidemiological, and genetic data. The phylogeny trees from Surveillance Reports of the WHO Influenza Centre for each H1N1 vaccine strain showed that the vaccine strain is frequently not only the strain at the top of a large clade but also the strain producing the majority of the clade offspring (see Supplementary Text). Thus, taking sequences of WHO vaccine as templates would give satisfying results most of the time. However, relying on WHO vaccine strains may not be fully warranted as new strains may also emerge from other circulating strains, or in emergent cases when the WHO vaccine strain is not published. Alternative templates would also be feasible as our results suggested in Fig. 2G that both New Caledonia/20/1999 and Solomon Islands/3/2006 templates work well in 2007–2008. Second example comes from the similar results from both the most abundant strain of A/California/07/2009 and the second abundant strain of A/Ohio/07/2009, as Supplementary Table S3 shows. Thus the template choosing is important but not super-sensitive. As the antigenic sequences of an influenza subtype are often similar during a circulation period, the final ranking of our model is expected to be robust for a substantial degree of template variations.

Currently identifying the right mutants from the candidate list is highly challenging, as it may be related to not only the fitness of each mutant but also the community population and even regional environment and social reporting system. However, our model provides a likely list of mutated HA sequences in future. Despite of the long list, they might form a few antigenic clusters. As being reported in previous studies, influenza virus evolves its antigenicity by population^18^. In addition to the traditional models correlating HA sequences with antigenicity map^19^,^20^, Liu *et al*. has recently developed latest PREDEC tools to calculate the antigenicity clusters based on HA sequences and showed good correlation with influenza circulation^5^,^21^. Given the likely list of future HA sequences, predicting new antigenicity cluster may become theoretically possible in future. It is noted that our model is based on continuous genetic evolution instead of antigenic evolution. According to Liu’s work^21^, our validation window covers two distinct phylogeny lineages (one before and one after 2009 swine-origin influenza virus) and three different antigenic clusters in major northern sphere (NE99, SO06 and CA09). From Fig. 2G, we can see that our work can perform well until 2009 when new lineage emerged. While during 2006–2008, the prediction from template CA09 can still maintain high performance even a new antigenic cluster SO06 formed. Although the validation time is limited because of the data collection, our model would be applicable to circulation period without genetic evolutional shift.

In conclusion, our study indicates that the temporal antigenic sequences can be leveraged to predict the mutating distribution of new antigenic sites in near future and provides an model to effectively make such predictions for the A/H1N1 and A/H3N2 viruses. In this paper, we divided the epitope areas into antigenic sites due to the limitation of computational power. Predicting epitope changes as a whole is theoretically feasible once the computational resources are available. As the assumption of this model is based on continuous genetic evolution, the model is currently limited to seasonal flu. Future improvements include a refined mutation matrix and dominance calculation incorporating mutational correlation between different antigenic sites as well as multiple templates. Coupled with the advanced antigenicity estimation, these predictions could help to target a group of upcoming epitopes that will be potentially dominant in the future rather than a single possibility. Given the rapid accumulation of high-resolution and high-coverage genomic data, our strategy may also be useful in anticipating the evolution of other highly mutagenic RNA viruses.

## Materials and Methods

### Data resources

All data was downloaded from the NCBI Influenza Virus Resources database^22^. For H1N1, training data covers 1593 HA sequences including 892 unique ones dated by Dec. 31, 2008, and testing data covers 11715 HA sequences dated from Jan. 1999 to Dec. 2014. For H3N2, training data contains 5278 HA sequences (1968–2011), while testing data contains 5915 HA sequences dated from Jan 2002 to Dec. 2014.

## Construction of a mutation model for HA antigenic sites. 

### Transition matrix of HA antigen

200 representative sequences were randomly chosen thrice from the 892 sequences with at least one sequence per year and at least one sequence per country. Multiple sequence alignment (MSA) was performed using Clustalw2^23^. Maximum parsimony (MP) phylogeny trees were generated by PHYLIP^24^, because the mutation rate is not constant here while MP was reported to be not sensitive to nucleotide substitution^25^. The nucleotide substitutions at four-fold degeneration sites were counted under neutral evolution. Then mutational probability was calculated among paired nucleotides as the number of substitutions divided by the corresponding total number of four-fold degeneration sites^26^. Finally, the nucleotide transition matrix was generated as an average. The transition matrix for amino acids was derived as below example: given a triplet code of ATC, its probability changing to ATT is


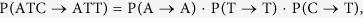


where *P* indicates the mutation probabilities among nucleotides.

### Mutation simulation of a given HA antigenic site

Given a template sequence in a specific antigenic sites, mutational sequences were randomly generated according to the specific probabilities of transition matrix. To balance the computational cost and coverage of potential mutations, 10^9^ simulations were performed for each antigenic site as a pool under neutral selection. This simulation was done 100 times before further ranking and filtering. For each simulation, we only used the antigenic site sequence from template rather than the whole HA1 sequence.

## Re-ranking the top abundant mutants by dominance likeliness. 

The dominance likeliness of a mutated antigenic sequence consists of historically adaptive ability (PSSM score^27^) and theoretical abundance in simulating results.

Set 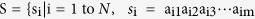, 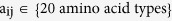, 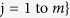 as MSA profile of N simulated sequences. N means the total number of simulated sequences in TOP 100 ranking list before sequence redundancy being removed. Set 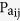 as the probability that amino acid 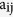 appears in position *j* of S, 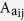 as the background frequency of 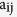
observed in whole HA sequences. 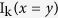 is a binary number that equals 1 if x = y, otherwise 0. 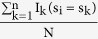 actually represents the relative amount of a unique sequence (top 1 to top 100) in the total number of simulated N results. The 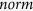 function is to adjust all values into range [0,1]. The dominance likeliness (DL) for 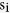 is calculated as follows:


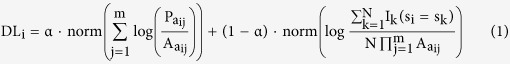


where the first part represents PSSM score of an antigenic mutant and the second part represents the theoretical abundance. In the second part, 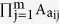 at the bottom is intended to delete amino acids preference, for the same purpose with the PSSM score part. The parameter 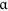 was artificially optimized by testing different values from 0 to 1 at step size of 0.05, and eventually 0.65 was chosen for H1N1 and 0.4 for H3N2. Noting the significant difference of both sequence accumulation and host-transferring before and after 2009, two PSSMs are introduced for H1N1. The pre-2009 PSSM is derived from sequence data from 1918 to 1999, whereas the post-2009 one is obtained from data reported merely in 2009.

## Evaluation parameters. 

### *Type coverage* was calculated as


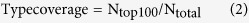


N_top100_ – the number of antigenic types correctly predicted in top 100 simulated mutant list; N _total_ – the total number of observed antigenic site types collected in corresponding period. An antigenic type is defined as one unique sequence of antigenic site such as Ca1, after redundancy being removed from multiple strain sequences reported.

### *Strain coverage* was calculated as


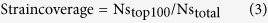


Ns_top100_ the number of observed strains carrying those successfully captured antigenic sites; Ns_total_ the total number of reported strains observed in the corresponding period.

An example to calculate the type coverage and strain coverage is illustrated in the Supplementary Text.

## Additional Information

**How to cite this article**: Xu, H. *et al*. Predicting the Mutating Distribution at Antigenic Sites of the Influenza Virus. *Sci. Rep*. **6**, 20239; doi: 10.1038/srep20239 (2016).

## Supplementary Material

Supplementary Information

## Figures and Tables

**Table 1:** Rank combination of dominant epitopes observed every year from 1999 to 2014.

**Year**	**Epitope with Relative abundance**^a^	**Rank combination**^b^	**Year**	**Epitope with Relative abundance**^a^	**Rank combination**^b^
1999^c^	54.55%	1,1,1,1,19	2007^d^	46.03%	1,2,2,1,6
2000^c^	30.68%	11,1,1,1,1	2008^d^	28.65%	1,1,2,1,--
	17.05%	11,1,1,1,11	2009^e^	57.09%	2,1,1,1,1
2001^c^	51.15%	1,1,1,18,1		18.48%	1,1,1,1,1
2002^c^	31.25%	1,1,1,18,19	2010^e^	44.49%	2,1,1,1,1
	18.75%	1,1,1,18,1		15.66%	2,1,1,1,24
2003^c^	46.94%	11,1,1,78,27	2011^e^	53.07%	2,1,1,1,24
	34.69%	11,1,1,1,1	2012^e^	65.80%	2,1,1,1,24
2004^c^	50.00%	11,1,1,1,1	2013^e^	43.55%	2,1,1,39,24
	25.00%	11,1,1,1,19		19.41%	2,1,1,1,24
2005^c^	77.78%	11,1,1,1,1	2014^e^	84.91%	2,1,1,39,24
2006^c^	28.32%	11,1,1,1,1			

^a^The relative abundance of epitopes (combined by five antigenic sites) are generated from epitope sequence variation in reported strains every year. Dominant epitopes are listed with relative abundance above 15% in the yearly reported data during 1999–2014.

^b^The predicted combination rank of five sites are for Ca1, Ca2, Cb, Sa, Sb, respectively. Only mutants falling within top100 are recorded, otherwise represented by ‘--’.

^c^The combination rank during 1999–2006 are predicted by template A/New Caledonia/20/1999.

^d^The combination rank during 2007–2008 are predicted by template A/Solomon Islands/3/2006.

^e^The combination rank during 2009-2014 are predicted by template A/California/7/2009.
