# Supplementary material for: Predicting the Mutating Distribution at Antigenic Sites of the Influenza Virus
Source: Sci Rep. 2016 Feb 3;6:20239. doi: 10.1038/srep20239 (PMC4738307; doi:10.1038/srep20239)
Supplement: Supplementary Information [file srep20239-s1.pdf]

Predicting the Mutating Distribution at Antigenic Sites of the Influenza Virus

Hongyang Xu, Yiyan Yang, Shuning Wang, Ruixin Zhu, Tianyi Qiu, Jingxuan Qiu, Qingchen Zhang, Li Jin, Yungang He, Kailin Tang & Zhiwei Cao

Supplementary Text

Surveillance Reports from WHO Influenza Centre

The phylogeny trees containing H1N1 vaccine strains can be found via links below.
Information for the vaccines and related biological products advisory committee 2001 Jan 30-31:
http://www.fda.gov/ohrms/dockets/ac/01/slides/3680s1_03.pdf;
http://www.fda.gov/ohrms/dockets/ac/01/briefing/3680b1_02.pdf
Information for the vaccines and related biological products advisory committee 2008 Feb 20-21:
http://www.fda.gov/ohrms/dockets/ac/08/briefing/2008-4348b2-1.pdf;
http://www.fda.gov/ohrms/dockets/ac/08/briefing/2008-4348B2-3.pdf

Positions of antigenic sites in HA epitope

For A/H1N1, below positions were extracted as 5 antigenic site according to literation:
Ca1: (169-173, 206-208, 238-240), Ca2 (140-145, 224-225), Cb (78-83), Sa (128-129, 156-160, 162-167), Sb (187-200).
For A/H3N2, amino acids positions of below were extracted as 5 antigenic site according to literation: A(122,124,131,133,137,143-146) ; B (155,156,158,160,164,188,189,193,196,197);
C (50,53,54,276,278); D (172,174,201,207,213,217,230,244) and E (62,75,82,83,260,262).

An example to illustrate the calculation of type coverage and strain coverage

Type coverage indicates the percentage of antigenic types being successfully predicted by the top 100 list among the total type of non-redundant antigenic sites. While strain coverage evaluates the spread width and distribution of antigenic sequences among all reported strains before removing redundancy.
For example, with site Sb of the A/New Caledonia/20/1999 strains as template, there are total of 99 HA sequences reported during 1999-2000 and 10 unique types after redundancy merge. Among the 10 types, 4 sequences are successfully predicted by our mutant profile (top 100), which cover 78 HA sequences. In this case, *type coverage* = 4/10 (40.00%), *strain coverage* = 78/99 (78.79%).

Supplementary Tables

Table S1. Detailed prediction results for the four template strains throughout the whole time span in 1999 to 2014.

(1). With A/Beijing/262/1995 strain (GenBank accession number ACF41867) as template strain

Period	Antigenic	Observed	Observed types in	Type	Strain

	Sites	types	top100 [a]	coverage [b]	coverage [b]
1999-2000	Ca1	8	3***	37.50%	78.79%
	Ca2	4	2***	50.00%	96.97%
	Cb	4	3***	75.00%	96.97%
	Sa	10	1*	10.00%	13.13%
	Sb	10	1*	10.00%	2.02%
	Average [c]			36.50%	57.58%
2001-2002	Ca1	6	4***	66.67%	95.92%
	Ca2	4	1*	25.00%	95.92%
	Cb	4	4***	100.00%	100.00%
	Sa	11	1*	9.09%	2.72%
	Sb	7	1*	14.29%	0.68%
	Average			43.01%	59.05%
2003-2004	Ca1	3	1**	33.33%	96.49%
	Ca2	2	1**	50.00%	98.25%
	Cb	1	1***	100.00%	100.00%
	Sa	3	0	0.00%	0.00%
	Sb	7	0	0.00%	0.00%
	Average			36.67%	58.95%
2005-2006	Ca1	8	1*	12.50%	93.57%
	Ca2	10	1*	10.00%	78.95%
	Cb	6	3**	50.00%	98.25%
	Sa	14	0	0.00%	0.00%
	Sb	17	0	0.00%	0.00%
	Average			14.50%	54.15%
2007-2008	Ca1	14	1*	7.14%	94.82%
	Ca2	24	2	8.33%	32.63%
	Cb	15	11***	73.33%	99.41%
	Sa	35	1	2.86%	0.47%
	Sb	63	1	1.59%	0.12%
	Average			18.65%	45.49%
2009-2010	Ca1	46	1	2.17%	4.84%
	Ca2	63	2	3.17%	0.02%
	Cb	35	4*	11.43%	4.99%
	Sa	92	1	1.09%	0.26%
	Sb	102	0	0.00%	0.00%
	Average			3.57%	2.02%
2011-2012	Ca1	20	0	0.00%	0.00%
	Ca2	24	0	0.00%	0.00%
	Cb	12	0	0.00%	0.00%
	Sa	41	0	0.00%	0.00%
	Sb	27	0	0.00%	0.00%

	Average			0.00%	0.00%
2013-2014	Ca1	16	0	0.00%	0.00%
	Ca2	20	0	0.00%	0.00%
	Cb	13	0	0.00%	0.00%
	Sa	29	0	0.00%	0.00%
	Sb	18	0	0.00%	0.00%
	Average			0.00%	0.00%

(2). With A/New Caledonia/20/1999 strain (GenBank accession number ACF41878) as template strain

**Period**	**Antigenic sites**	**Observed types**	**Observed types in top100** [a]	**Type coverage** [b]	***Strain* coverage** [b]
1999-2000	Ca1	8	3***	37.50%	78.79%
	Ca2	4	4***	100.00%	100.00%
	Cb	4	3***	75.00%	96.97%
	Sa	10	7***	70.00%	96.97%
	Sb	10	4**	40.00%	78.79%
	Average			64.50%	90.30%
2001-2002	Ca1	6	4***	66.67%	95.92%
	Ca2	4	4***	100.00%	100.00%
	Cb	4	4***	100.00%	100.00%
	Sa	11	7**	63.64%	97.28%
	Sb	7	5***	71.43%	92.52%
	Average			80.35%	97.14%
2003-2004	Ca1	3	1**	33.33%	96.49%
	Ca2	2	2***	100.00%	100.00%
	Cb	1	1***	100.00%	100.00%
	Sa	3	3***	100.00%	100.00%
	Sb	7	5***	71.43%	96.49%
	Average			80.95%	98.60%
2005-2006	Ca1	8	1*	12.50%	93.57%
	Ca2	10	6***	60.00%	97.66%
	Cb	6	3***	50.00%	98.25%
	Sa	14	9***	64.29%	95.91%
	Sb	17	7**	41.18%	89.47%
	Average			45.59%	94.97%
2007-2008	Ca1	14	1*	7.14%	94.82%
	Ca2	24	7*	29.17%	94.23%
	Cb	15	11***	73.33%	99.41%
	Sa	35	23***	65.71%	94.70%
	Sb	63	7*	11.11%	62.19%
	Average			37.29%	89.07%

2009-2010	Ca1	46	1	2.17%	4.84%
	Ca2	62	3*	4.84%	3.30%
	Cb	35	4	11.43%	4.99%
	Sa	92	11*	11.96%	4.79%
	Sb	102	1	0.98%	0.02%
	Average			6.28%	3.59%
2011-2012	Ca1	20	0	0.00%	0.00%
	Ca2	24	0	0.00%	0.00%
	Cb	12	0	0.00%	0.00%
	Sa	41	0	0.00%	0.00%
	Sb	27	0	0.00%	0.00%
	Average			0.00%	0.00%
2013-2014	Ca1	16	0	0.00%	0.00%
	Ca2	20	0	0.00%	0.00%
	Cb	13	0	0.00%	0.00%
	Sa	29	0	0.00%	0.00%
	Sb	18	0	0.00%	0.00%
	Average			0.00%	0.00%

(3). With A/Solomon Island/3/2006 strain (GenBank accession number ABU99109) as template strain

**Period**	**Antigenic Sites**	**Observed types**	**Observed types in top100** [a]	**Type coverage** [b]	**Strain coverage** [b]
2007-2008	Ca1	14	10***	71.43%	99.41%
	Ca2	24	9**	37.50%	95.05%
	Cb	15	3.15*	21.00%	98.25%
	Sa	35	23***	65.71%	94.70%
	Sb	63	1	1.59%	36.75%
	Average			39.45%	84.83%
2009-2010	Ca1	46	5*	10.87%	4.99%
	Ca2	62	8*	12.90%	4.89%
	Cb	35	2*	5.71%	4.91%
	Sa	92	11*	11.96%	4.79%
	Sb	102	0	0.00%	0.00%
	Average			8.29%	3.92%
2011-2012	Ca1	20	0	0.00%	0.00%
	Ca2	24	0	0.00%	0.00%
	Cb	12	0	0.00%	0.00%
	Sa	41	0	0.00%	0.00%
	Sb	27	0	0.00%	0.00%
	Average			0.00%	0.00%
2013-2014	Ca1	16	0	0.00%	0.00%
	Ca2	20	0	0.00%	0.00%

	Cb	13	0	0.00%	0.00%
	Sa	29	0	0.00%	0.00%
	Sb	18	0	0.00%	0.00%
	Average			0.00%	0.00%

(4). With A/California/07/2009 strain (GenBank accession number ACP44189) as template strain

Period	Antigenic Sites	Observed types	Observed types in top100 [a]	Type coverage [b]	Strain coverage [b]
2009-2010	Ca1	47	9^{*}	19.15%	92.45%
	Ca2	62	27^{**}	43.55%	94.02%
	Cb	35	23^{***}	65.71%	94.64%
	Sa	92	41^{**}	44.57%	94.25%
	Sb	102	27.5^{**}	26.96%	94.46%
	Average			39.99%	93.96%
2011-2012	Ca1	20	2^{*}	10.00%	87.13%
	Ca2	24	18^{***}	75.00%	98.39%
	Cb	12	11^{***}	91.67%	99.82%
	Sa	41	26^{**}	63.41%	97.86%
	Sb	27	8.5^{**}	31.48%	94.55%
	Average			54.31%	95.55%
2013-2014	Ca1	16	3^{*}	18.75%	95.87%
	Ca2	20	14^{***}	70.00%	98.87%
	Cb	13	12^{***}	92.31%	99.92%
	Sa	29	13^{**}	44.83%	95.95%
	Sb	18	4^{*}	22.22%	92.64%
	Average			49.62%	96.65%

[a] *p-value was calculated to testify whether the enrichment of observed antigenic sites types including in TOP 100 mutants list is of statistical significance or not. The result is significant at * p-value<0.05, ** p-value<0.01, *** p-value<0.001.*

[b] *Type coverage standard deviation<0.05; Strain coverage standard deviation<0.005.*

[c] *The averaged values of the combined 5 antigenic sites.*

Table S2. Reported time for different templates in 2009 and the clades they belong to.

GenBank accession number	Strain name	Reported time	Clade
ACP41105	A/California/04/2009	2009/04/01	Top 1 abundant clade
ACP44189	A/California/07/2009	2009/04/09	Top 1 abundant clade

ACR09372	A/Mexico/3955/2009	2009/04/02	Top 38 abundant clade
ACQ76386	A/Ohio/07/2009	2009/04/24	Top 2 abundant clade

Table S3. Performance of different template sequences in 2009.

(1). With A/Mexico/3955/2009 strain (GenBank accession number ACR09372) as the template strain

Period	Antigenic Sites	Observed types	Observed types in top100 [a]	Type coverage [b]	Strain coverage [b]
2009-2010	Ca1	47	9**	19.15%	92.45%
	Ca2	62	10*	16.13%	6.12%
	Cb	35	0	0.00%	0.00%
	Sa	92	41**	44.57%	94.25%
	Sb	102	27.8*	27.25%	94.47%
2011-2012	Ca1	20	2*	10.00%	87.13%
	Ca2	24	3*	12.50%	2.14%
	Cb	12	0	0.00%	0.00%
	Sa	41	26**	63.41%	97.86%
	Sb	27	8.8*	32.59%	94.60%
2013-2014	Ca1	16	3*	18.75%	95.87%
	Ca2	20	3*	15.00%	92.80%
	Cb	13	0	0.00%	0.00%
	Sa	29	13**	44.83%	95.95%
	Sb	18	5*	27.78%	92.80%

Note: for Cb site, the original strain is XSTASS, X stands for unknown amino acid, so the program couldn't predict unknown amino acid

(2)With A/Ohio/07/2009 (GenBank accession number ACQ76386), the earliest one in the second most abundant clade in April, as template strain

Period	Antigenic Sites	Observed types	Observed types in top100 [a]	Type coverage [b]	Strain coverage [b]
2009-2010	Ca1	47	25**	53.19%	94.23%
	Ca2	62	27*	43.55%	94.02%
	Cb	35	23**	65.71%	94.64%
	Sa	92	41**	44.57%	94.25%
	Sb	102	27.6*	27.06%	94.47%
2011-2012	Ca1	20	13**	65.00%	99.11%

	Ca2	24	18^{***}	75.00%	98.39%
	Cb	12	11^{***}	91.67%	99.82%
	Sa	41	26^{**}	63.41%	97.86%
	Sb	27	8.6^{*}	31.85%	94.57%
2013-2014	Ca1	16	15^{***}	93.75%	99.92%
	Ca2	20	14^{***}	70.00%	98.87%
	Cb	13	12^{***}	92.31%	99.92%
	Sa	29	13^{**}	44.83%	95.95%
	Sb	18	5^{*}	27.78%	92.80%

[a] *p-value was calculated to testify whether the enrichment of observed antigenic sites types including in TOP 100 mutants list is of statistical significance or not. The result is significant at * p-value<0.05, ** p-value<0.01, *** p-value<0.001.*

[b] *Type coverage standard deviation<0.05; Strain coverage standard deviation<0.005.*

Table S4. Potential variants profile of antigenic sites in future with the sequence in top 1 clade (the most abundant clade only in 2014) as template.

Top20 in Ca1	Top20 in Ca2	Top10 in Cb	Top50 in Sa	Top50 in Sb
INDKGTSREPG	PHAGAKRD	LSTASS	PNKKGNSPKLSQS	TTADQQSLYQNA
INDKGSSREPG	PHAGARRD	LSSASS	PNKKGNSPKLSKS	TSADQQSLYQNA
INDKETSREPG	PHAGAKRE	FSTASS	PNKKGNSPKLSRS	TIADQQSLYQNA
INDKGTSHEPG	PHAGEKRD	LITASS	PNKKGNSPKLSES	TMADQQSLYQNA
INNKGTSREPG	SHAGAKRD	LSTARS	PNKKGNSPNLSQS	TNADQQSLYQNA
INDRGTSREPG	PHAGANRD	LSTASP	PNRKGNSPKLSQS	TPADQQSLYQNA
INDKRTSREPG	PHAGAKRG	LPTASS	PNKKGDSPKLSQS	TAADQQSLYQNA
MNDKGTSREPG	PHAGAKRN	LSTTSS	PNKKGNLPKLSQS	TRADQQSLYQNA
INDKGTSREPR	PHDGAKRD	LFTASS	PNKKGNSPRLSQS	TTVDQQSLYQNA
VNDKGTSREPG	PHAGVKRD	LSMASS	PNKKGNSPKLSHS	TTADQQTLYQNA
INDKGTSRGPG	PHAGTKRD		PDKKGNSPKLSQS	TTADQQSLYRNA
INDKGTSRDPG	LHAGAKRD		PNEKGNSPKLSQS	TTADQQSLYQTA
INDKGTSRKPG	PYAGAKRD		PNKKGNSPKFSQS	TTADQQSLYHNA
INDKGMSREPG	PHTGAKRD		PNKKGNSPMLSQS	TTADQQSLYQNE
INDKGASREPG	PHAEAKRD		PNKKGNSPKLSQP	NTADQQSLYQNA
TNDKGTSREPG	PHAGAMRD		PNKKGNSPKLRQS	TTTDQQSLYQNA
IHDKGTSREPG	PQAGAKRD		PNKQGNSPKLSQS	TTADQKSLYQNA
INDMGTSREPG	PHVGAKRD		PNKKGNPPKLSQS	MTADQQSLYQNA
INDKGTSKEPG	PRAGAKRD		PNMKGNSPKLSQS	STADQQSLYQNA
FNDKGTSREPG	PHAGAQRD		PNKKGKSPKLSQS	TTADQQSIYQNA
			PNKKGSSPKLSQS	TKADQQSLYQNA
			PNKKENSPKLSQS	TTADQQSLYQDA
			PNKEGNSPKLSQS	TTADQQSFYQNA
			PNKKGNSPKLSQF	TTADQQSLYQNV
			PNKKGNSPKLPQS	TTADQQPLYQNA

		PNKKGNSPKLSPS	TTADQQRLYQNA
		PSKKGNSPKLSQS	TTADQQSLYQSA
		PNKKGNSPKPSQS	TTANQQSLYQNA
		PHKKGNSPKLSQS	TTADQQSPYQNA
		PNKKGNSPKMSQS	TTADQQSMYQNA
		PNKKGNSPKISQS	TTADQRSLYQNA
		PNKKGNSPELSQS	ATADQQSLYQNA
		PNKMGNSPKLSQS	TTSDQQSLYQNA
		PNKKRNSPKLSQS	TTADQQSLHQNA
		PNKRGNSPKLSQS	TTADQQFLYQNA
		PNKKGNSPKLNQS	ITADQQSLYQNA
		PNKKGNSPKLFQS	TTADQQGLYQNA
		PNKKGNSPQLSQS	TTADRQSLYQNA
		PNKKGHSPKLSQS	TTDDQQSLYQNA
		PNQKGNSPKLSQS	PTADQQSLYQNA
		PNKKGNSPTLSQS	TTADQQSLYQNT
		PKKKGNSPKLSQS	TTADQQSLCQNA
		PNKKGNFPKLSQS	TTAGQQSLYQNA
		PNKNGNSPKLSQS	TTADQQSLYQHA
		PNKKGNSPKLSQY	TTADQQNLYQNA
		PIKKGNSPKLSQS	TTGDQQSLYQNA
		PNKKGNSPKLSQR	TTADQQCLYQNA
		PNKKGNSPKLSLS	TTAVQQSLYQNA
		PNKKGNSPKLTQS	TTADQQSLYQKA
		PNKKGNSPKLCQS	TTADQQALYQNA

Note: From the data we observed, some antigenic sites can be correctly predicted within Top 20 even Top 10. Therefore, top potential list with different lengths are given here.

Table S5. H3N2 combination rank of dominant epitopes observed every year from 2002-2014.

Year	Top dominant strains	Relative abundance [a]	Combination rank [b]	Template strain
2002	1	78.69%	--,--,2,1,6	A/Fujian/411/2002
2003	1	65.99%	1,1,1,1,1	A/Fujian/411/2003
2004	1	45.43%	6,1,1,1,1	A/Wellington/01/2004
			1,--,1,1,1	A/California/07/2004
2005	1	34.53%	6,46,1,1,1	A/Wellington/01/2004
			1,--,1,1,1	A/California/07/2004
2005	2	22.10%	72,27,1,1,1	A/Wellington/01/2004
			9,--,1,1,1	A/California/07/2004
2005	3	20.72%	6,1,1,1,1	A/Wellington/01/2004
			1,--,1,1,1	A/California/07/2004
2006	1	43.13%	2,11,1,1,1	A/Wisconsin/67/2005

	2	38.75%	2,11,12,1,1	A/Wisconsin/67/2005
2007	1	66.41%	2,11,12,1,1	A/Wisconsin/67/2005
2008	1	70.11%	1,1,1,1,1	A/Brisbane/10/2007
	2	17.60%	1,1,1,1,--	A/Brisbane/10/2007
2009	1	30.21%	1,1,1,34,1	A/Perth/16/2009
	2	24.11%	2,36,1,1,6	A/Perth/16/2009
2010	1	46.31%	2,1,12,6,6	A/Perth/16/2009
	2	36.44%	2,1,1,1,6	A/Perth/16/2009
2011	1	40.68%	1,11,12,6,1	A/Victoria/361/2011
	2	21.33%	9,11,1,1,1	A/Victoria/361/2011
2012	1	39.76%	9,11,10,1,1	A/Victoria/361/2011
	2	21.03%	1,11,12,6,1	A/Victoria/361/2011
	3	15.10%	9,11,1,1,1	A/Victoria/361/2011
2013	1	83.55%	9,11,10,1,1	A/Victoria/361/2011
2014	1	52.15%	9,11,10,1,1	A/Victoria/361/2011
	2	22.01%	--,--,10,1,1	A/Victoria/361/2011

[a] *The relative abundance of epitopes (combined by five antigenic sites) are generated from each HA sequence reported. Dominant epitopes with relative abundance > 15% are listed every year from 2002-2014.*

[b] *The predicted combination rank of five sites are for A, B, C, D, E, respectively. Only mutants within top100 are recorded, otherwise represented by '--'.*

Table S6. Detailed prediction results for the seven H3N2 template strains throughout the whole time span in 2002 to 2014.

(1). With A/Fujian/411/2002 (H3N2)-like as template strain

Period	Antigenic Sites	Observed types	Observed types in top100 [a]	Type coverage [b]	Strain coverage [b]
2002-2003	A	10	6***	62.80%	77.78%
	B	11	6***	54.55%	66.43%
	C	10	8***	84.60%	99.78%
	D	6	6***	100.00%	100.00%
	E	10	7***	70.00%	99.42%
2004-2005	A	12	4***	35.67%	83.44%
	B	17	3***	17.65%	60.53%
	C	13	11***	84.62%	99.71%
	D	11	11***	100.00%	100.00%
	E	10	8***	80.00%	99.56%
2006-2007	A	13	2***	15.38%	92.32%
	B	7	1**	14.29%	1.87%

	C	11	7***	63.64%	99.17%
	D	7	7***	100.00%	100.00%
	E	10	7***	70.00%	97.93%
2008-2009	A	15	2***	13.33%	59.71%
	B	15	1**	6.67%	0.33%
	C	11	6***	54.55%	98.90%
	D	9	9***	100.00%	100.00%
	E	13	9***	69.23%	92.65%
2010-2011	A	21	2***	9.52%	75.06%
	B	14	0	0.00%	0.00%
	C	18	7***	38.89%	95.40%
	D	8	7***	87.50%	99.62%
	E	17	10***	58.82%	93.07%
2012-2013	A	24	2***	8.33%	21.88%
	B	17	0	0.00%	0.00%
	C	16	4***	25.00%	29.20%
	D	12	8***	66.67%	99.69%
	E	12	7***	58.33%	99.63%
2014	A	8	2***	25.00%	58.85%
	B	10	0	0.00%	0.00%
	C	5	0	0.00%	0.00%
	D	4	2***	50.00%	99.04%
	E	9	4***	44.44%	96.17%

(2). With A/Wellington/01/2004 (H3N2)-like as template strain

**Period**	**Antigenic Sites**	**Observed types**	**Observed types in top100** [a]	**Type coverage** [b]	**Strain coverage** [b]
2004-2005	A	12	4***	35.33%	83.43%
	B	17	10***	61.65%	98.74%
	C	13	11***	84.62%	99.71%
	D	11	11***	100.00%	100.00%
	E	10	8***	80.00%	99.56%
2006-2007	A	13	2***	15.38%	92.32%
	B	7	2***	28.57%	97.30%
	C	11	7***	63.64%	99.17%
	D	7	7***	100.00%	100.00%
	E	10	7***	70.00%	97.93%

2008-2009	A	15	2***	13.33%	59.71%
	B	15	2***	13.33%	50.93%
	C	11	6***	54.55%	98.90%
	D	9	9***	100.00%	100.00%
	E	13	9***	69.23%	92.65%
2010-2011	A	21	2***	9.52%	75.06%
	B	14	1**	7.14%	0.08%
	C	18	7***	38.89%	95.40%
	D	8	7***	87.50%	99.62%
	E	17	10***	58.82%	93.07%
2012-2013	A	24	2***	8.33%	21.88%
	B	17	0	0.00%	0.00%
	C	16	4***	25.00%	29.20%
	D	12	8***	66.67%	99.69%
	E	12	7***	58.33%	99.63%
2014	A	8	2***	25.00%	58.85%
	B	10	0	0.00%	0.00%
	C	5	0	0.00%	0.00%
	D	4	2***	50.00%	99.04%
	E	9	4***	44.44%	96.17%

(3). With A/California/07/2004 (H3N2)-like as template strain

**Period**	**Antigenic Sites**	**Observed types**	**Observed types in top100** [a]	**Type coverage** [b]	**Strain coverage** [b]
2004-2005	A	12	7***	58.33%	94.85%
	B	17	2***	12.00%	8.94%
	C	13	11***	84.62%	99.71%
	D	11	11***	100.00%	100.00%
	E	10	8***	80.00%	99.56%
2006-2007	A	13	10***	76.92%	98.96%
	B	7	0	0.57%	0.07%
	C	11	7***	63.64%	99.17%
	D	7	7***	100.00%	100.00%
	E	10	7***	70.00%	97.93%
2008-2009	A	15	10***	66.67%	95.28%
	B	15	0	0.27%	0.01%
	C	11	6***	54.55%	98.90%

	D	9	9***	100.00%	100.00%
	E	13	9***	69.23%	92.65%
2010-2011	A	21	11***	50.10%	96.62%
	B	14	0	0.00%	0.00%
	C	18	7***	38.89%	95.40%
	D	8	7***	87.50%	99.62%
	E	17	10***	58.82%	93.07%
2012-2013	A	24	10***	42.58%	96.74%
	B	17	0	0.00%	0.00%
	C	16	4***	25.00%	29.20%
	D	12	8***	66.67%	99.69%
	E	12	7***	58.33%	99.63%
2014	A	8	2***	25.00%	58.85%
	B	10	0	0.00%	0.00%
	C	5	0	0.00%	0.00%
	D	4	2***	50.00%	99.04%
	E	9	4***	44.44%	96.17%

(4). With A/Wisconsin/67/2005 (H3N2)-like as template strain

Period	Antigenic Sites	Observed types	Observed types in top100 [a]	Type coverage [b]	Strain coverage [b]
2006-2007	A	13	6***	46.15%	96.68%
	B	7	2***	28.57%	95.85%
	C	11	7***	63.64%	99.17%
	D	7	7***	100.00%	100.00%
	E	10	7***	70.00%	97.93%
2008-2009	A	15	8***	53.33%	95.06%
	B	15	2***	13.33%	50.71%
	C	11	6***	54.55%	98.90%
	D	9	9***	100.00%	100.00%
	E	13	9***	69.23%	92.65%
2010-2011	A	21	8***	38.10%	96.23%
	B	14	1**	7.14%	0.08%
	C	18	7***	38.89%	95.40%
	D	8	7***	87.50%	99.62%
	E	17	10***	58.82%	93.07%
2012-2013	A	24	7***	29.34%	95.42%
	B	17	0	0.00%	0.00%
	C	16	4***	25.00%	29.20%

2014	D	12	8***	66.67%	99.69%
	E	12	7***	58.33%	99.63%
	A	8	3***	37.50%	62.20%
	B	10	0	0.00%	0.00%
	C	5	0	0.00%	0.00%
	D	4	2***	50.00%	99.04%
	E	9	4***	44.44%	96.17%

(5). With A/Brisbane/10/2007 (H3N2)-like as template strain

**Period**	**Antigenic Sites**	**Observed types**	**Observed types in top100** [a]	**Type coverage** [b]	**Strain coverage** [b]
2008-2009	A	15	10***	66.67%	95.28%
	B	15	8***	53.33%	51.59%
	C	11	10***	90.91%	99.89%
	D	9	9***	100.00%	100.00%
	E	13	9***	69.23%	92.65%
2010-2011	A	21	10***	49.90%	96.60%
	B	14	2***	14.29%	0.15%
	C	18	15***	83.33%	99.10%
	D	8	7***	87.50%	99.62%
	E	17	10***	58.82%	93.07%
2012-2013	A	24	10***	42.92%	96.66%
	B	17	0	0.00%	0.00%
	C	16	12***	75.00%	99.63%
	D	12	8***	66.67%	99.69%
	E	12	7***	58.33%	99.63%
2014	A	8	2***	25.00%	58.85%
	B	10	1***	10.00%	65.07%
	C	5	2***	40.00%	96.65%
	D	4	2***	50.00%	99.04%
	E	9	4***	44.44%	96.17%

(6). With A/Perth/16/2009 (H3N2)-like as template strain

**Period**	**Antigenic Sites**	**Observed types**	**Observed types in top100** [a]	**Type coverage** [b]	**Strain coverage** [b]
2010-2011	A	21	9***	42.86%	98.04%
	B	14	9***	64.29%	98.79%
	C	18	15***	83.33%	99.10%
	D	8	7***	87.50%	99.62%

	E	17	11***	64.71%	93.90%
2012-2013	A	24	6***	25.00%	95.60%
	B	17	13***	76.47%	99.38%
	C	16	12***	75.00%	99.63%
	D	12	8***	66.67%	99.69%
	E	12	7***	58.33%	99.44%
2014	A	8	3***	37.50%	59.33%
	B	10	4***	40.00%	93.78%
	C	5	2***	40.00%	96.65%
	D	4	2***	50.00%	99.04%
	E	9	4***	44.44%	96.17%

(7). With A/Victoria/361/2011 (H3N2)-like as template strain

**Period**	**Antigenic Sites**	**Observed types**	**Observed types in top100** [a]	**Type coverage** [b]	**Strain coverage** [b]
2012-2013	A	24	10***	41.92%	96.61%
	B	17	3***	17.65%	98.26%
	C	16	12***	75.00%	99.63%
	D	12	8***	66.67%	99.69%
	E	12	7***	58.33%	99.63%
2014	A	8	3***	37.50%	59.33%
	B	10	2***	20.00%	65.55%
	C	5	2***	40.00%	96.65%
	D	4	2***	50.00%	99.04%
	E	9	4***	44.44%	96.17%

[a] *p-value was calculated to testify whether the enrichment of observed antigenic sites types including in TOP 100 mutants list is of statistical significance or not. The result is significant at * p-value<0.05, ** p-value<0.01, *** p-value<0.001.*

[b] *Type coverage standard deviation<0.05; Strain coverage standard deviation<0.005.*

Supplementary Figures

Figure S1

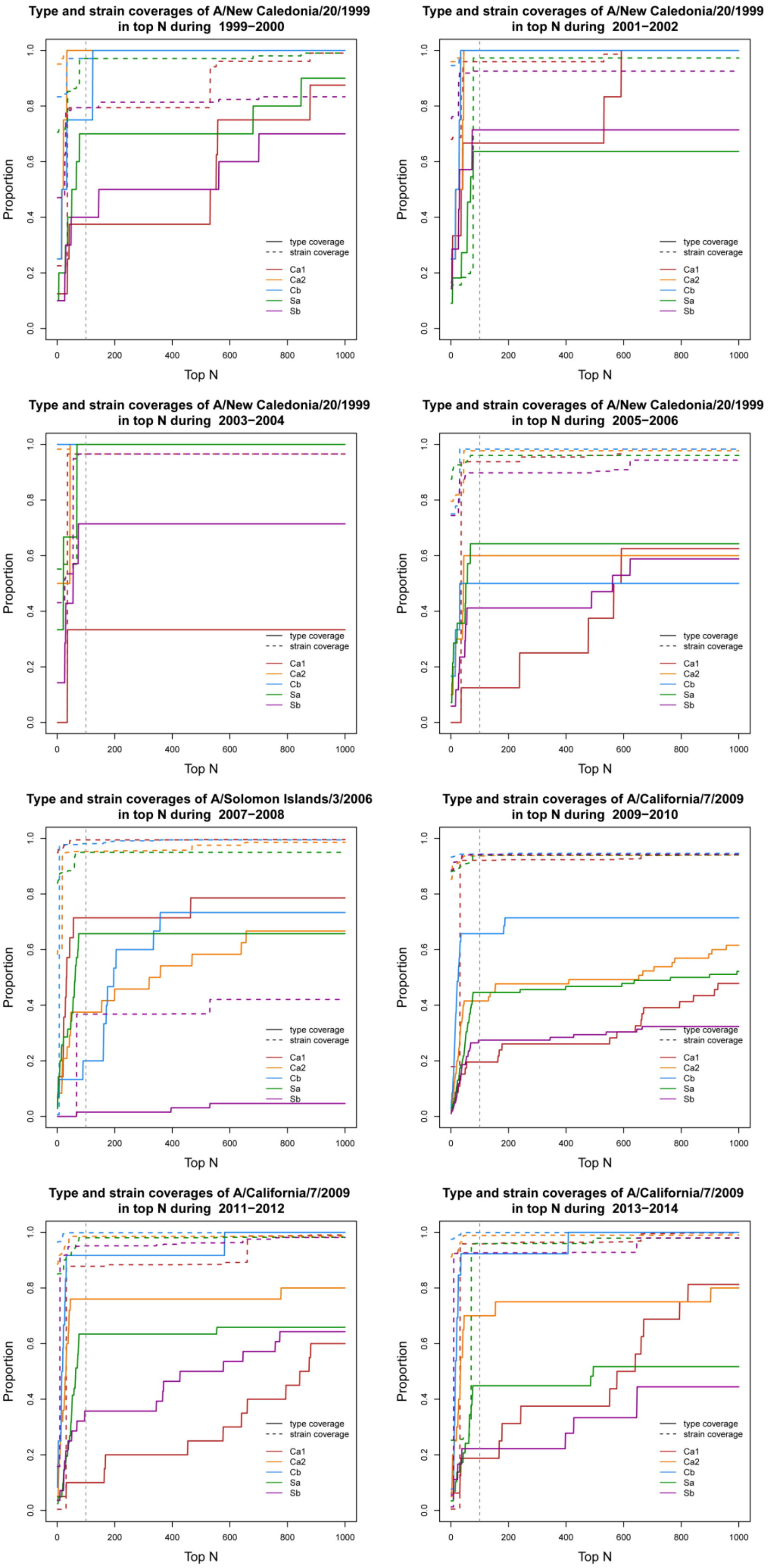

Figure S1. The type coverage and strain coverage of H1N1 calculated with different cutoffs. The top 100 is indicated by a gray dashed line in vertical. Results of each template in five antigenic sites are shown in each subgraph. The Y-axis on the left indicates the type coverage (solid line) and strain coverage (dashed line) in proportion.

Figure S2

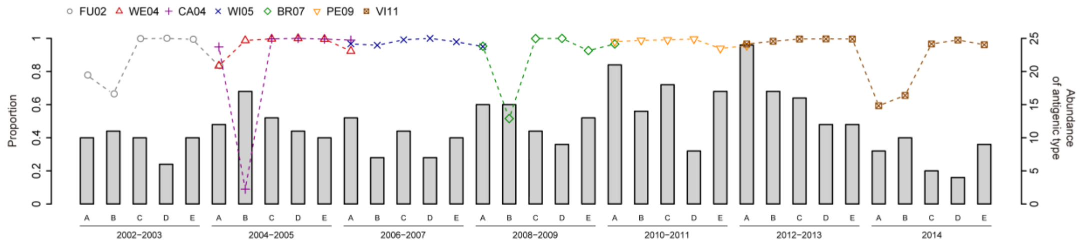

Figure S2. The strain coverage of our model for H3N2 during 2002-2014, with five antigenic sites and seven consecutive templates. The Y-axis on the left indicates the strain coverage in proportion. FU02, WE04, CA04, WI06, BR07, PE09 and VI11 are short for A/Fujian/411/2002, A/Wellington/01/2004, A/California/07/2004, A/Wisconsin/67/2005, A/Brisbane/10/2007, A/Perth/16/2009 and A/Victoria/361/2011 respectively.

Figure S3

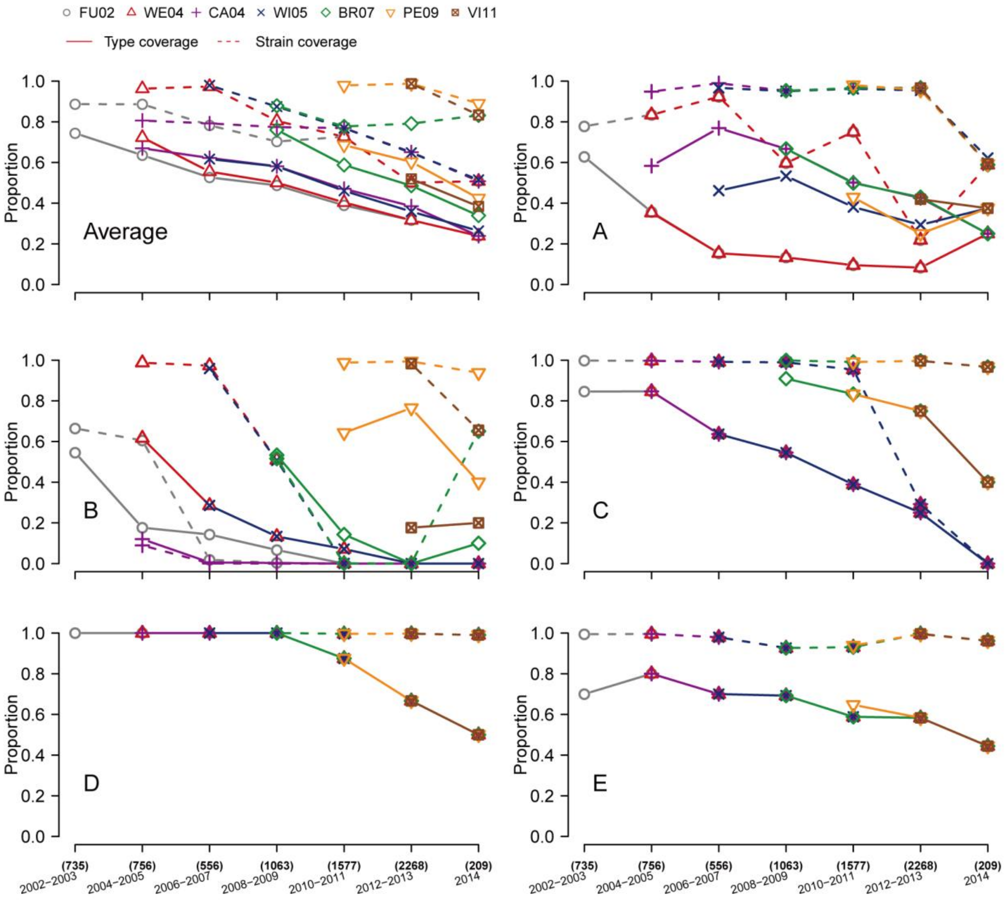

Figure S3. The type coverage and strain coverage for H3N2 during the entire validation period of 2002-2014. Results of individual antigenic sites as well as the averaged are shown with seven different template indicated. The Y-axis on the left indicates the type coverage (solid line) and strain coverage (dashed line) in proportion. FU02, WE04, CA04, WI06, BR07, PE09 and VI11 are short for A/Fujian/411/2002, A/Wellington/01/2004, A/California/07/2004, A/Wisconsin/67/2005, A/Brisbane/10/2007, A/Perth/16/2009 and A/Victoria/361/2011 respectively.
